# Impact of Glutenin/Gliadin Ratio and Maltodextrin on Structural and Functional Properties of Soy Protein Isolate–Wheat Gluten Protein Composite Gel

**DOI:** 10.3390/gels11110916

**Published:** 2025-11-16

**Authors:** Min Qu, Chang Ge, Sitong Li, Ying Zhu, Peixiu Jiang, Yuyang Huang, Bingyu Sun, Linlin Liu, Xiuqing Zhu

**Affiliations:** College of Food Engineering, Harbin University of Commerce, Harbin 150028, China; qumin777@126.com (M.Q.);

**Keywords:** soy protein isolate, wheat gluten protein, maltodextrin, composite gel properties

## Abstract

Enhancing the gel properties of soy protein isolate (SPI) is crucial for forming stable gel systems through interactions with other plant proteins and polysaccharides. This study investigated the contribution of different ratios of glutenin (Glu)/gliadin (Gli) and maltodextrin (MD) to SPI–wheat gluten protein (WGP) composite gels. SPI-WGP composite gels were prepared by varying the Glu/Gli ratio (0:10, 3:7, 4:6, 5:5, 6:4, 7:3, and 10:0) and adjusting the MD addition level (0, 2, 4, and 6%). Subsequently, the textural properties, water-holding capacity (WHC), rheological behavior, secondary structure, intermolecular forces, and microstructure of the composite gels were characterized. Results indicated that adding 4% MD with a Glu/Gli ratio of 4:6, compared with the SPI control group gel, the WHC, gel strength, and β-sheet content of the composite gel increased by 37.9%, 164.5%, and 30.6%, respectively. Hydrophobic interactions and hydrogen bonds became dominant after MD incorporation. Sodium dodecyl sulfate–polyacrylamide gel electrophoresis (SDS-PAGE) and scanning electron microscopy (SEM) confirmed that the two proteins interact with MD to form a supported, dense, and homogeneous gel system. Excess MD caused phase separation in the composite gel system, disrupting the gel structure. This study provides important references for the development and potential applications of SPI-WGP composite gels.

## 1. Introduction

Soy protein isolate (SPI) is nutritionally balanced, has good gelation and water absorption, and it is routinely regarded as a functional or non-functional “filler” in food processing. Consequently, it has become an important source of high-quality plant protein [1]. In general, SPI is folded and unfolded upon heating and forms aggregates through hydrogen bonds, van der Waals forces, and hydrophobic interactions, thereby forming a stable three-dimensional gel network structure [2]. Nonetheless, the formation of single SPI gel is easily restricted by poor mechanical properties, low solubility, and processing conditions [3]. The use of exogenous macromolecules such as proteins and carbohydrates to modify protein gel behavior and improve food processing quality and nutritional value [4] has become an important means to improve SPI gel properties.

Wheat gluten protein (WGP) is composed of glutenin (Glu) and gliadin (Gli) and possesses properties such as viscoelasticity and extensibility. Notably, its viscoelasticity is unique among plant proteins, which can promote the formation of a compact and continuous dual-protein elastic gel network with SPI during extrusion, thereby improving the quality of extrudates [5,6]. However, due to its lysine deficiency [7] and the presence of substantial hydrophobic groups, WGP has inadequate water solubility and gel properties, which limits its application in food [8]. The interaction between WGP and SPI can sustain the charged state of the protein surface and inhibit the expansion behavior of SPI gel during preparation, thereby improving gel strength [9]. The relationship between the changes in the structure and composition of WGP and its functional properties as well as the quality of wheat products has gradually attracted attention. For example, recombinant Glutelin with different ratios of Glu/Gli diminishes the viscosity, rheological properties, and gel strength of starch gels [10]. Barak et al. [11] found that the gliadin/glutenin (Gli/Glu) ratio was positively correlated with the hardness of breadcrumbs and exerted an influence on bread volume. Qu et al. [12] revealed that Gli and Glu contributed to the change in WGP gel behavior. Glu contributed to enhancing the elasticity of the WGP gel and strengthening the protein network skeleton, while Gli improved the strength and WHC of WGP, thereby enhancing the gel performance and optimizing the network structure.

The gelation and thickening ability of polysaccharides facilitates the interaction of protein molecules and bolsters the structural and functional properties of protein gels by interfering with the unfolding and aggregation of protein molecular chains [13]. This confers a superior performance on the composite gel formed by proteins and various polysaccharide groups [14,15]. Maltodextrin (MD) is a starch-derived oligosaccharide polymer with moderate sweetness. It is a linear long chain composed of D-glucose residues linked by α-1,4 glycosidic bonds and has a sturdy hydration ability and water-holding capacity [16]. While MD forms a protein–polysaccharide complex with its own carbonyl group and the amino group of the protein, it ameliorates the structure and texture of the composite gel by combining a large amount of hydroxyl groups with water [17]. Zhao C et al. [18] utilized MD in filling in the gel gap of SPI in producing a stable composite gel system. This composite gel structure assisted in improving the functional properties of protein emulsification [19], solubility, and thermal stability [20]. Notably, these studies have focused on the interaction between MD and SPI, while the synergistic effects of the Glu/Gli ratio and MD content on the structural transitions and gel network of SPI-WGP systems remain unreported.

In this study, changes in the gel strength, textural properties, rheological properties, and water-holding capacity, etc., of SPI-WGP composite gel under different Glu/Gli ratios and MD inclusion levels were investigated. This study also examined the structural changes induced on intermolecular force, gel electrophoresis, secondary structure, and the endogenous fluorescence spectrum. The purpose of this study was to elucidate the effect and interaction mechanism of the Glu and Gli ratio and MD inclusion levels on the structure and properties of SPI-WGP composite gel under heat induction and the contribution of Glu and Gli and MD to the properties of the SPI-WGP system. It is expected to provide theoretical references for the development and utilization of mixed raw materials (e.g., SPI combined with other plant proteins and polysaccharides) as plant-based meat alternatives and textured food matrices.

## 2. Results and Discussion

### 2.1. Textural Analysis

#### 2.1.1. Gel Strength Properties

**Figure 1:**
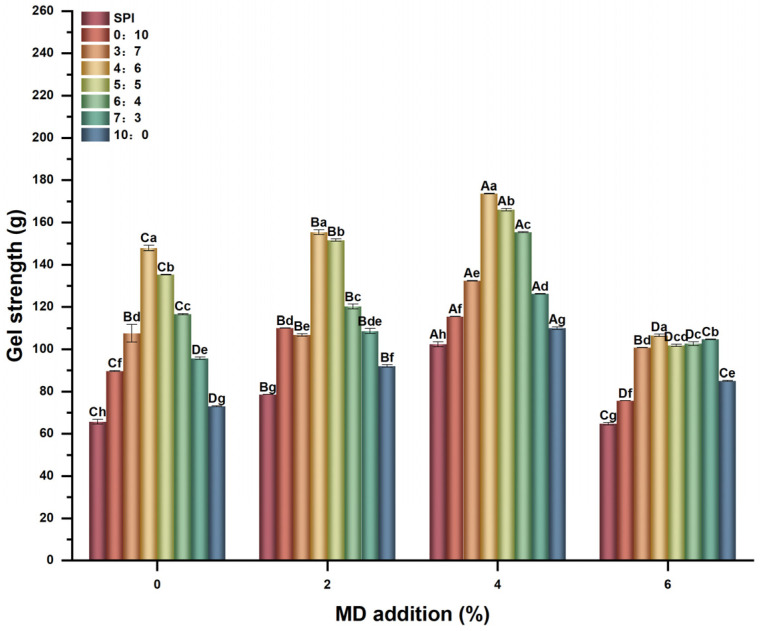
Changes in gel strength of SPI-WGP composite gel with different Glu/Gli ratios and MD inclusion levels. Different uppercase and lowercase letters indicate significant differences at *p* < 0.05 (*n* = 3).

Gel strength reflects the ability of protein to form a gel, which is related to the structure of the gel network [21]. As shown in Figure 1, with the increase in the proportion of Gli in Glu/Gli, the gel strength of SPI-WGP composite gel increased first and then decreased, reaching 147.86 g at 4:6, which was 125.3% higher than that of the single SPI control group, and then significantly increased (*p* < 0.05). This indicates that the filling of Gli can enhance the Glu chain, plasticize the gluten network structure, and promote the full combination of SPI with strong thermal gelation, thereby improving gel strength. Delcour et al. [22] also reported a similar phenomenon.

With the increase in MD addition, the gel strength of the composite gel showed a trend of first rising and then decreasing. When the Glu/Gli was 4:6 and MD addition was 4%, the maximum gel strength reached 173.64 g, which was 164.5% higher than that of the single SPI control group. MD can compose a protein–polysaccharide complex through carbonyl ammonia reaction, and its large number of hydroxyl groups can be combined with water to improve the structure and texture of the composite gel by changing the protein gelation behavior [17]. Zhao H et al. [23] observed that the presence of polysaccharides promoted the binding of protein molecules through steric hindrance effect, stimulated the interaction between protein–protein and protein–polysaccharide, and improved gel strength [24]. Thus, this indicates that the three-dimensional network structure formed by the mixing of Glu and Gli was the basis for the formation of higher gel strength with SPI. The addition of MD increased the stability of the Gli-Glu skeleton, promoted further aggregation of the SPI-Glu/Gli protein, and enhanced the gel strength of the composite gel.

Notably, the gel strength of SPI-WGP composite gels with high MD levels decreased simultaneously, especially at ratios 4:6~6:4. This indicates that excessive MD destroyed the interaction between proteins and the stability of the gel network, resulting in phase separation of the SPI-WGP composite gel and decreased gel strength [25]. This had a greater impact on SPI-WGP composite gels with high Gli content, that is, the interaction between the fillers was greater.

#### 2.1.2. Textural Properties

**Figure 2:**
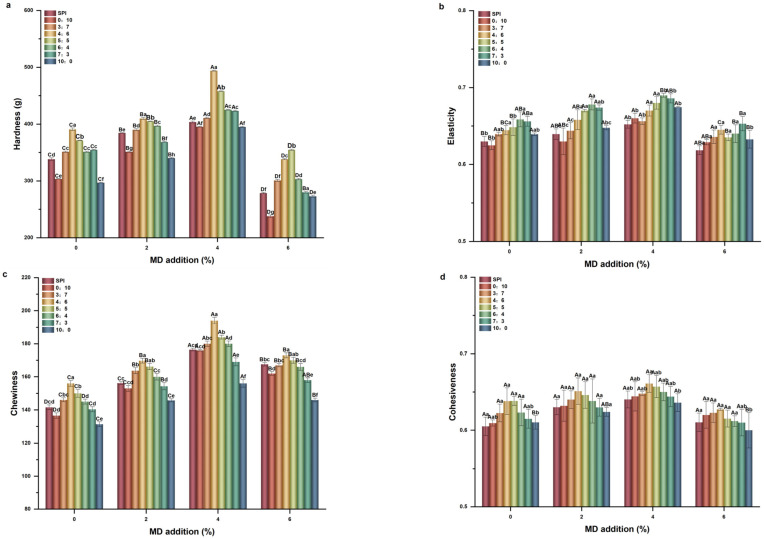
Changes in texture properties of SPI-WGP composite gel with different Glu/Gli ratios and MD inclusion levels. (**a**–**d**): Hardness, elasticity, chewability, and cohesion, respectively. Different uppercase and lowercase letters indicate significant differences at *p* < 0.05 (*n* = 3).

Effect of different Glu/Gli ratios and MD inclusion levels on the textural properties of the SPI-WGP composite gel was studied (Figure 2). Compared with the single SPI control group gel, the elasticity and cohesion of SPI-WGP composite gel increased slightly. The hardness and chewiness of the composite gel with the ratio of Glu/Gli of 3:7–6:4 increased, while the hardness and chewiness of the 10:0, 0:10 group decreased. With increased Gli, all studied textural properties of the SPI-WGP composite gel increased initially and then decreased, among which hardness and chewiness changed significantly. Hardness, chewiness, and cohesiveness were maximum at a ratio of 4:6 and elasticity was the largest at a ratio of 6:4. The gluten network formed by Gli and Glu was stronger than that of their binding ability with SPI. The inclusion of Glu provided a certain degree of elastic skeleton and enhanced the elasticity of the composite gel. A certain proportion of Gli could enhance the stability of the gel network structure, thereby improving the hardness, chewiness, and cohesiveness of the SPI-WGP composite gel.

With the increase in MD inclusion, all the studied textural properties of the composite gel increased initially and then decreased, while hardness and chewiness were the most affected (*p* < 0.05). The hardness, chewiness, and cohesiveness of the 4%, 4:6 group reached the maximum, which were 493.964 g, 194 g, and 0.661, respectively. The hardness and chewiness were significantly higher than those of the other groups (*p* < 0.05). Elasticity reached 0.69 at 6:4. It can be seen that the effect of Gli on the hardness and chewiness of the composite gel was greater than that of Glu. At the same time, MD further strengthened the skeleton structure of Glu, enhanced the elasticity of the composite gel, and improved the ability of Gli to stabilize the skeleton structure of Glu through non-covalent bonds, thereby improving the hardness, chewiness, and cohesiveness of the composite gel. This was consistent with the findings of Dekkers et al. [6]. The addition of polysaccharides facilitated the combination of SPI and WGP and the stability of the three-dimensional network of the gel, improving the hardness and chewiness of the composite gel. However, excessive MD reduced the texture properties of the SPI-WGP composite gel, especially the hardness, which may be due to the excessive filling of MD after hydration, resulting in the destruction of the protein–polysaccharide gel network and the reduction in hardness. Excessive glycosylation generated a decrease in its solubility, giving rise to a weakened gel strength, hardness, and WHC, which was similar to the findings of Ma et al. [26].

### 2.2. Rheological Properties

**Figure 3:**
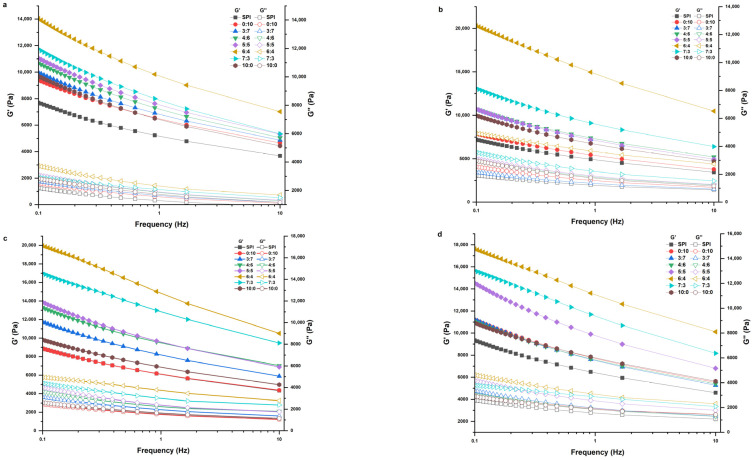
Changes in rheological properties of SPI-WGP composite gel with different Glu/Gli ratios and MD inclusion levels. (**a**–**d**): Modulus diagrams for MD inclusion levels of 0, 2, 4, and 6%, respectively.

Viscoelasticity of protein gels can be reflected by the storage modulus (G′) and loss modulus (G″) during frequency scanning, which are expressed as the elastic and viscous properties of the gel, respectively [27]. As shown in Figure 3, the G′, G″ values of all groups did not intersect, and G′ > G″, indicating that all gel samples were physical gels rather than covalent gels. Compared with the single SPI control group gel, the G′ and G″ values of the SPI-WGP composite gel increased. With increase in Gli, the G′, G″ values of the SPI-WGP composite gel increased initially, attaining the maximum at a ratio of 6:4, and then decreased. Notably, the G′ and G″ values in the group with higher Gli content were both higher than those in the group with higher Glu content. Glu has more intramolecular and intermolecular disulfide bonds (S-S), which are prone to aggregation and have good elasticity. At the same time, it provides more binding sites, which increases the probability of SPI cross-linking and enhances the elasticity [28].

MD further increased the G′ and G″ values of all SPI-WGP composite gels. With the increase in MD, the G′ and G″ values of the composite gels increased initially and then decreased, which was prominent at high Glu inclusion. The values of G′ and G″ attained the maximum at 4% MD and a Glu/Gli ratio of 6:4, which were 20,312.0 Pa and 4954.9 Pa. This may be due to the embedding of MD, further narrowing the gap of the polymer network, which was beneficial to the improvement of the resistance to external force and elastic properties of the gel in the rheological test [29]. Wang et al. [14] also reported similar findings, revealing that the addition of *Mesona chinensis* polysaccharide enhanced the elastic behavior of the SPI gel and promoted the formation of a tighter gel network. In addition, studies have shown that the enhancement of gel elasticity is also related to the enhanced interaction between polysaccharides and proteins [30]. The results revealed that the increase in the Glu proportion promoted the formation of the gel network skeleton, and the addition of MD further promoted the aggregation of Glu and cross-linking with SPI, strengthened the gel network, and enhanced the viscoelasticity. The reason for the change in gel viscoelasticity may be due to the fact that, with the increase in MD content, the content of hydrophobic and hydrophilic groups (acetyl and carboxyl) increases, resulting in an increase in entanglement points in the system, the enhancement of hydrogen bonds between proteins and intermolecular interactions, a more solid gel network structure, and the enhancement of mechanical stability of the composite gel [31]. However, excessive Gli, Glu, and MD will reduce the rheological properties of SPI-WGP composite gels. This is because when the concentration of the substance is too high, the gel formed by the heat-induced method will have uneven molecular aggregation, resulting in shrinkage of the gel structure and destruction of the gel network continuity, which in turn affects the viscoelastic behavior of the gel system [32]. This is consistent with the trend of elasticity in the textural properties under 2.1.2.

### 2.3. Water-Holding Capacity (WHC)

**Figure 4:**
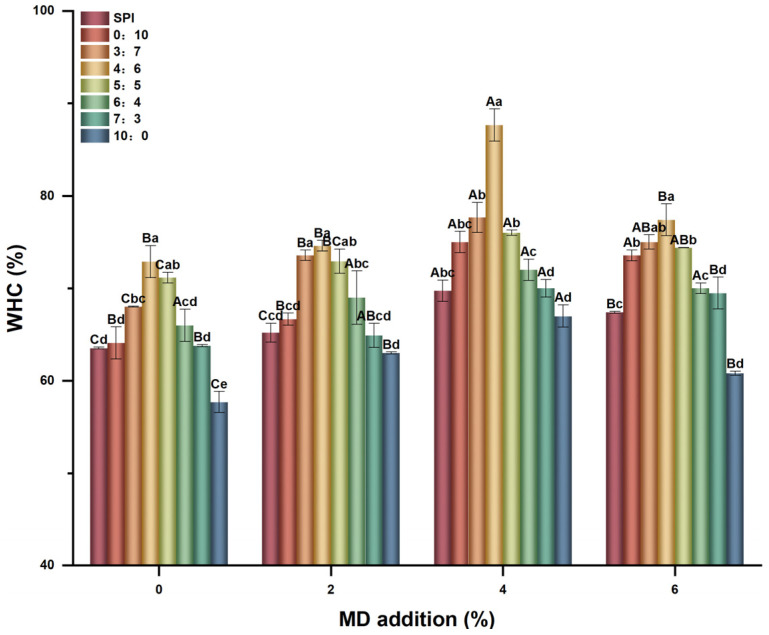
Changes in WHC of SPI-WGP composite gel at different Glu/Gli ratios and MD inclusion levels. Different uppercase and lowercase letters indicate significant differences at *p* < 0.05 (*n* = 3).

WHC refers to the ability of proteins to retain water after being heated to form a three-dimensional network gel structure. As shown in Figure 4, when MD was not added, with the increase in the proportion of Gli, the WHC showed a trend of increasing first and then decreasing. Among them, the 4:6 group was the largest, reaching 72.9%, which was 14.7% higher than that of the single SPI control group. And the high proportion of Gli has a greater increase. The hydrophobic groups in Gli and SPI molecules made them have better water-binding capacity than Glu [33]. Therefore, the high ratio of Gli ameliorated the water-retention capacity of the composite gel, while the high hydrophobicity of Glu impaired the water-binding capacity of the system and reduced the WHC.

MD further increased the WHC of all SPI-WGP composite gels. The increase in MD inclusion first led to an increase in WHC followed by a decrease, especially the composite gels with a high proportion of Gli, which had a significantly increased WHC (*p* < 0.05). At 4% MD, the WHC of the group with a Glu/Gli ratio of 4:6 reached 87.6%, which was 37.9% higher than that of the single SPI control group. This may be due to the high hydration capacity of MD, which facilitated synergy between Gli and SPI, thereby improving the ability of protein to retain water and increasing the WHC. Thus, the polysaccharide chain was able to improve gel WHC as a continuous phase formed by the embedded protein, which is consistent with the study of Zhao C et al. [18]. Notably, the 10:0 group showed the lowest values among all SPI and Glu/Gli groups. Although MD supplementation improved performance, it remained the lowest among all groups.

Therefore, the effect of Gli on the WHC of the composite gel is greater than that of Glu, MD can significantly increase the WHC of the composite gel with a high proportion of Gli (*p* < 0.05), and excessive MD (6%) will reduce the WHC. This finding coincides with that of Li L et al. [34], who reported that a congruent amount of dandelion root polysaccharide significantly increased the WHC of whey protein isolate gel, while excessive addition showed the opposite results.

### 2.4. Molecular Forces

**Figure 5:**
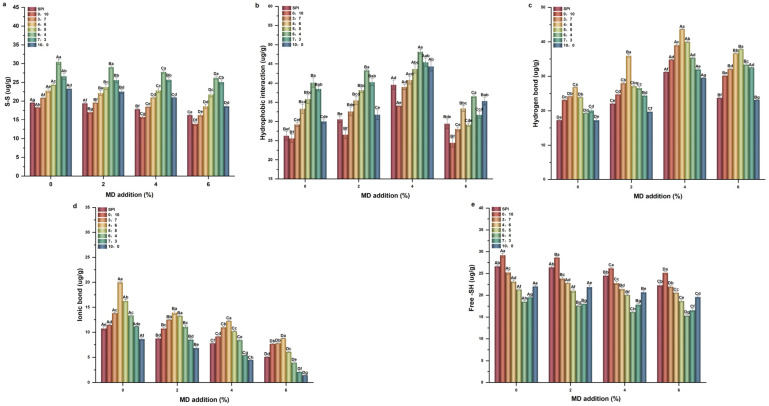
Changes in intermolecular force of SPI-WGP composite gel at different Glu/Gli ratios and MD inclusion levels. (**a**–**e**): The content of disulfide bonds, hydrophobic interactions, hydrogen bonds, ionic bonds, and free -SH, respectively. Different uppercase and lowercase letters indicate significant differences at *p* < 0.05 (*n* = 3).

Effect of different Glu/Gli ratios and MD inclusion levels on the intermolecular force in the SPI-WGP composite gel was studied (Figure 5). At 0% MD addition, the ratio of each bond in the SPI and SPI-WGP composite gel was as follows: hydrophobic interaction > S-S > hydrogen bond > ionic bond. The group with a Glu/Gli ratio of 6:4 exhibited the highest contents of S-S bonds and hydrophobic interactions, while the 4:6 group had the highest contents of hydrogen bonds and ionic bonds. Notably, the group with a Glu/Gli ratio of 10:0 showed the highest free -SH content. After adding MD, the free -SH content decreased continuously. Meanwhile, the S-S bond content slightly decreased, the contents of hydrophobic interactions and hydrogen bonds first increased and then decreased, and the ionic bond content decreased consistently. At 4% MD content and a Glu/Gli ratio of 4:6, the hydrogen bond content peaked (43.69 ug/g); at a Glu/Gli ratio of 6:4, hydrophobic interactions peaked (48.16 ug/g). At 6% MD addition and a Glu/Gli ratio of 10:0, the ionic bond content reached the minimum (1.43 ug/g). Ultimately, the dominant intermolecular forces in the system shifted from “hydrophobic interactions + S-S” to “hydrophobic interactions + hydrogen bonds”. Since hydrophobic interactions remained the predominant force throughout, the entropy increase (ΔS > 0) derived from hydrophobic interactions served as the core driving force for this transition, making the entire process entropy-driven [35]. The results indicated that MD significantly affected the hydrophobic interactions and disulfide bonds of Glu, while having a substantial impact on the hydrogen bonds and ionic bonds of Gli. This can be attributed to the addition of Gli and Glu, which provided more hydrogen bonding forces, S-S, and hydrophobic interactions through hydration and thermally exposed hydrophobic groups [23]. Although MD is a neutral polysaccharide, its abundant hydroxyl groups can form hydrogen bonds with the charged groups of proteins, creating a “hydration layer” on the protein molecular surface. This layer physically shields the charged sites, reducing the probability of ionic bond formation and thus leading to a decrease in ionic bond content [36]. This is the same as the changing trend of 2.3 WHC, indicating that there is the presence of hydrophilic groups on the surface of Gli under heat induction, the ability to form hydrogen bonds is powerful, and the ability to bind water is strong. The results of Li C et al. [37] revealed that the addition of coix seed starch to gluten gel significantly increased the contribution of the hydrogen bond and hydrophobic interaction, while the contribution of the ionic bond and S-S decreased, which was consistent with the conclusion of this current study. However, due to the inherent limitations of the method used to detect intermolecular forces in this study, it cannot effectively determine the S-S cross-linking of Glu. This results in a significantly low extraction rate of Glu aggregates and an underestimation of their binding strength. Detailed experimental data are provided in the Supplementary File S1.

### 2.5. Endogenous Fluorescence Spectrum

**Figure 6:**
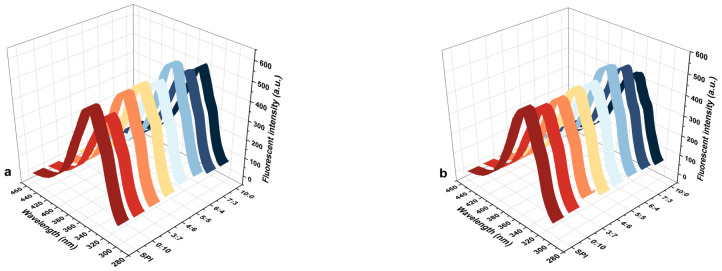

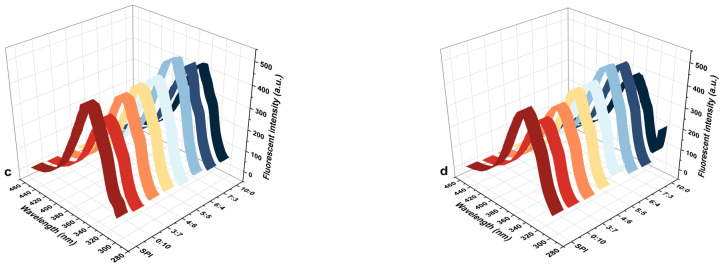
Changes in endogenous fluorescence spectrum of SPI-WGP composite gel at different Glu/Gli ratios and MD inclusion levels. (**a**–**d**): Spectrum plots for MD addition of 0, 2, 4, and 6%, respectively.

Endogenous fluorescence spectroscopy is one of the methods to detect the change in the protein tertiary structure. The fluorescence intensity indicates the transfer of the aromatic amino acid Trp residue between the protein core and the polar environment. When the hidden hydrophobic group is exposed, the polarity of the microenvironment around the Trp residue decreases and the hydrophobicity increases. The exposure degree of Trp residue also reflects the looseness of protein conformation [38]. MD decreased the fluorescence intensity of the SPI-WGP composite gel (Figure 6). λ_max_ of the group with a high proportion of Gli was red-shifted, and the red-shift degree at ratio 4:6 was the largest. λ_max_ was blue-shifted at the high Glu proportion, and the blue shift induced by the group with a ratio of 6:4 was the largest. However, the fluorescence intensity at the ratio of 6:4 was always the highest. During the heat-induced gelation process, the protein molecules were unfolded at high temperatures, thereby exposing Trp residues. After cooling, the internal Trp was exposed to a polar environment, thereby reducing the fluorescence intensity, and the λ_max_ was red-shifted [38]. Due to the galore Trp of Glu, it was easier to be buried in a hydrophobic environment than that of Gli, which had a high fluorescence intensity. When SPI was cross-linked with Glu, λ_max_ blue-shifted [39].

MD reduced the fluorescence intensity of the SPI-WGP composite gel. With the increase in the inclusion level, the λ_max_ of the SPI gel and composite gel with the high Gli was red-shifted, and the wavelength increased first and then decreased. The λ_max_ of the composite gel with high Glu blue-shifted, and the wavelength decreased first and then increased. The exposure level of Trp residues increased continuously, accompanied by a gradual loosening of the protein conformation. When the addition amount was 4%, the red shift in the 4:6 group was the largest, and the blue shift in the composite gel with a ratio of 6:4 was the largest. When the addition level of MD increased to 6%, the exposure level of Trp residues decreased slightly, and the protein conformation tightened moderately while remaining looser than that without the MD addition. The addition of MD led to the non-covalent binding of protein hydrophobic groups in the system, and the number of Trp residues exposed to the hydrophilic environment increased, thereby reducing the fluorescence intensity [40]. MD resulted in a red shift in the Trp residue λ_max_ of SPI and Gli and a blue shift in the Trp residue of Glu due to partial masking [37]. Notably, the Trp exposure level in the group with a Glu/Gli ratio of 6:4 was higher than that in the groups with extreme ratios (0:10, 10:0), indicating a more significant loosening of the protein conformation at this specific ratio. These results demonstrate that the conformational stability of the composite gel protein exhibited a trend of first decreasing and then slightly recovering with the increase in MD addition. This may explain that MD promotes the enhancement of hydrophobic interactions with Glu in 2.4.

### 2.6. Fourier Infrared Spectra (FTIR)

Infrared spectroscopy was used to detect the structural changes in proteins and the interaction between proteins and polysaccharides in the mixed system. The wide band at 3200–3400 cm^−1^ reflects the change in intermolecular and intramolecular hydrogen bond strength [41]. The peak of the SPI-WGP composite gel was lower than that of the single SPI control group gel, and an increase in Gli inclusion led to an initial decrease in the peak, with the lowest attained at a ratio of 4:6, followed by an increase. It showed that Gli induced the formation of more hydrogen bonds, which enhanced the interaction between WGP and SPI, and the hydrogen bond force in Gli is reflected more in Section 2.4. The increase in MD made the peaks of each group decrease first and then increase but reduced the peaks of the composite gels, reaching the lowest at the 4% and 4:6 groups. This indicates that the addition of MD enhanced the stretching vibration of hydroxyl hydration, promoted the binding of protein (especially Gli) to water, which was presumably dominated by hydrogen bond-mediated non-covalent interactions, and then made the gel system more compact. Chu et al. [42] found that the addition of starch shortened the SPI gel peak and enhanced the hydrogen bonding. Excess MD led to the increase in peak intensity, which may be due to the reaction of MD with the protein to form a water-insoluble polymer, which hindered the partial binding of the sample to water and weakened the hydrogen bond interaction between the components [43].

**Figure 7:**
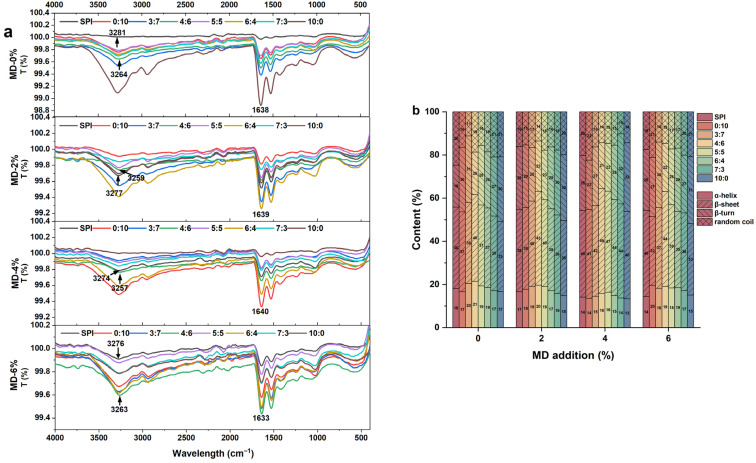
Changes in infrared spectrum of SPI-WGP composite gel at different Glu/Gli ratios and MD inclusion levels. (**a**) Spectrogram, (**b**) percentage of secondary structure.

The proportion of β-sheet in the secondary structure of all the histones was relatively high, accounting for approximately 31–49% (Figure 7b). The α-helix and β-sheet contents in gels containing only Gli and Glu, respectively, were lower than the composite gels. An increase in Gli led to an initial increase in α-helix and β-sheet contents, with the highest contents at a ratio of 4:6, and then they decreased. However, the addition of MD reduced the content of the α-helix and increased the content of the β-sheet in all groups. The content of β-sheet reached the highest value of 48.6% when adding the 4%, 4:6 group, which was 30.6% higher than that of the single SPI control group. The transition from α-helix to β-sheet is the basis of protein gelation, and the addition of MD promotes this transition, especially in the group with the high proportion of Gli. This may be due to MD, which interfered with the unfolding and aggregation of protein polypeptides, promoted the cross-linking of SPI, WGP, and MD, and increased the degree of gelation [37]. Zheng et al. [44] highlighted that MD competed with protein for water molecules, produced protein–water hydrogen bonds, and enhanced the fluidity of the system to form a β-sheet conformation. Since the α-helix conformation was responsible for the chain folding state of Gli, the reaction of the carbonyl group in MD with the amino group of the α-helix in Gli intensified this folding state and produced a tighter structure [45]. It can be seen from the figure that the change in the β-sheet is the most significant, which determines that the rigid structure of the gel is stronger and the mechanical strength is stronger. Among the secondary structures, β-sheets contribute the most significantly to mechanical reinforcement [46]. In addition, the β-turn content of the composite gel in the group with a high proportion of Glu was higher, and the addition of MD further increased the content of β-turn, which was a sign of protein aggregation [47]. Glu and MD promoted the aggregation and cross-linking between the components of the composite gel.

Accordingly, Gli played a decisive role in the secondary structure and hydrogen bond interaction of SPI-WGP composite protein gel. An appropriate amount of MD increased the β-turn content in Glu and enhanced cross-linking between protein and polysaccharide. It also facilitated the conversion of α-helix to β-sheet in SPI and Gli and upgraded the degree of gelation. This is consistent with the results of the gel strength in 2.1.1.

### 2.7. Protein Molecular Weight

**Figure 8:**
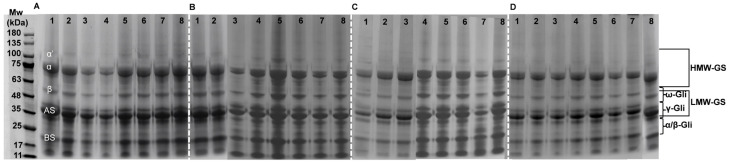
Changes in molecular weight of SPI-WGP composite gel at different Glu/Gli ratios and MD inclusion levels. (**A**–**D**) MD additions of 0, 2, 4, and 6%, respectively; 1–8 Glu/Gli ratio of 0:0~10:0.

SPI mainly contains acidic subunits (AS, 38 kDa), basic subunits (BS, 17 kDa), α subunits (67 kDa), α’ subunits (71 kDa), and β subunits (50 kDa). Glu contains HMW-GS (66–130 kDa) and LMW-GS (36–44 kDa); Gli contains ω (39–55 kDa), γ (38–42 kDa), and α/β (28–40 kDa) subunits. Figure 8 is the electrophoresis map of the composite gel with different proportions of Glu/Gli and MD addition. SPI and 0:10~5:5 groups contained more low-molecular-weight components. Without MD addition, high Glu content (5:5–10:0) significantly increased high-molecular-weight components (*p* < 0.05). Stacking phenomena appeared in higher-molecular-weight bands, exceeding 140 kDa [48]. At 2% MD addition, high Glu content (5:5–10:0) further increased the molecular weight. However, at 4% MD addition, the molecular weight decreased in all groups except the pure SPI group, where it increased substantially. Notably, the high Glu ratio group (5:5–10:0) showed a significant decrease (*p* < 0.05), falling below 100 kDa, accompanied by increased banding of the Gli α/β subunit (28–38 kDa). At 6% MD addition, the molecular weight decreased in the SPI group to levels similar to the single SPI control group, while changes in other groups were minimal. Thus, lower MD (2%) promoted the aggregation of high Glu ratios (5:5–10:0) toward higher molecular weights, while increased MD induced the depolymerization of high-molecular-weight fractions. This indicates that MD interacts with HMW-GS in Glu and α/β (28–38 kDa) subunits in Gli. Wang et al. [49] suggested that this may result from Maillard reaction-formed complexes between polysaccharides and proteins. This may be due to the high HMW-GS content in Glu, as well as HMW-GS being aggregated by disulfide bonds. MD destroyed the S-S between polymer molecules, which was consistent with the change in S-S content in 2.4. Yang et al. [29] depicted that polysaccharides could not interact with peanut protein through covalent bonds, which was related to the change in surface hydrophobicity of peanut protein during the gelation process.

### 2.8. Microscopic Morphology (2000×)

**Figure 9:**
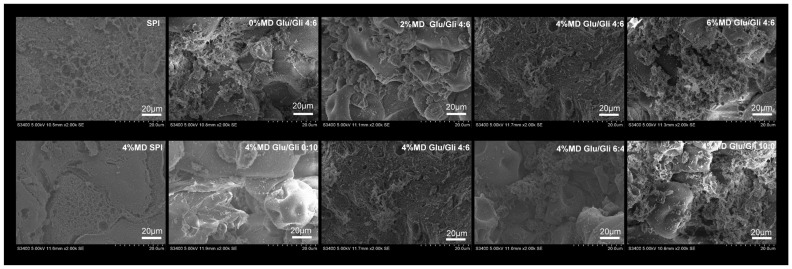
Changes in microstructure of SPI-WGP composite gel at different Glu/Gli ratios and MD inclusion levels (2000×).

The SPI surface of the control showed a certain continuous network structure, but the network was weak and uneven (Figure 9). After mixing SPI with MD, most of the network structure disappeared, and the surface structure tended to be smooth and dense. Interestingly, when the Glu/Gli ratio was 4:6, SPI-WGP appeared to have an obvious “strong” network and filling structure. An increase in MD gradually filled the network cavity, and the spherical SPI was embedded in the network structure. When MD was 4%, the network was completely filled, continuous, and uniform, and SPI was completely covered. The network was broken and large holes appeared, then the filler was exposed when the MD was excessive (6%). With a change in the Glu/Gli ratio and an increase in Glu, there was no obvious network on the surface of the mixed system, SPI was bonded and gradually presented a network structure, and SPI was completely covered. Glu/Gli ratios higher than 6:4 led to disruption of the network structure until a large cavity was generated and SPI was re-exposed. This revealed that the SPI gel had a weak network structure, and a certain proportion of Glu/Gli provided strong network support. Under the mediation of MD, SPI-MD-Gli aggregates were filled in the mixed network structure with Glu as the main framework to form a supported dense and uniform gel system. Hu et al. [50] also drew such a conclusion, and the results concurred with the finding in Section 2.1.1. This indicates that a more compact microscopic network structure can support higher gel strength. Therefore, in the SPI-WGP composite gel, Glu formed a network skeleton, and Gli, SPI, and MD were cross-linked and filled in the network skeleton. When the proportion of the four components was appropriate, the SPI-WGP composite gel structure was the most compact and stable, otherwise, competitive hydration occurred, resulting in the destruction of the WGP network and the composite gel structure.

### 2.9. The Mechanism of the Influence of Different Proportions of Glu/Gli and MD on the Properties of SPI-WGP Composite Gel

**Figure 10:**
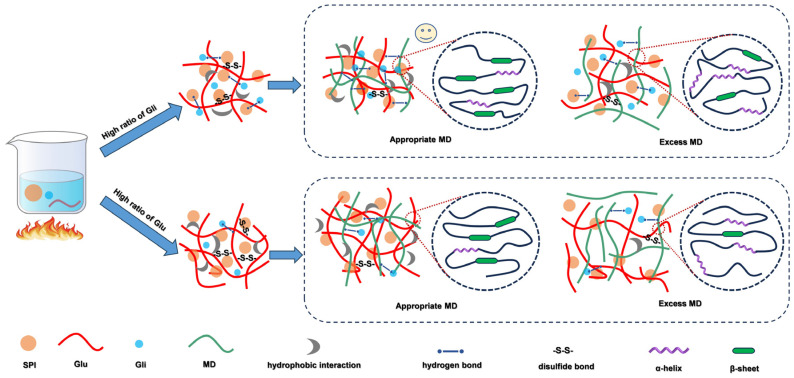
Mechanism of the interaction between SPI-WGP and MD on the properties of composite gel.

The different structural and functional properties of Gli and Glu led to different roles in the formation of protein gels, and the intervention of MD affected the gel behavior of proteins (Figure 10). Under water-hydrolysis conditions, WGP formed a polymer network structure and formed a composite protein gel network with SPI, in which Glu provided a fibrous skeleton support. SPI and Gli were embedded in the skeleton structure of Glu as a “filler” to form a new gel structure. The ratio of Glu determined the quantity of network skeletons, while the ratio of Gli influenced filling efficiency, gel strength, and water retention. Excessive Glu might result in overabundant skeleton structures, leading to insufficient filling and weakened gel properties. Conversely, excessive Gli might cause insufficient skeletons, resulting in overly compact filling that might even overflow, thereby reducing gel strength, water retention, and elasticity. This is in agreement with Qu et al. [12]. The main intermolecular forces that maintain the SPI-WGP composite gel are hydrophobic interactions and disulfide bonds, and the change in the ratio of Glu and Gli affects the structure of the composite gel.

After adding MD, the primary intermolecular forces maintaining composite gels were now dominated by hydrophobic interactions and hydrogen bonds. The hydroxyl groups in MD participated in hydrogen bond formation within protein systems, altering the intermolecular forces of protein gels and promoting the transformation of the α-helix to β-sheet. This enhanced both the hydrophobic interactions between SPI and Glu, as well as the hydrogen bond content of Gli, thereby providing additional spatial and structural support for cross-linking between Gli and SPI. Non-covalent binding between proteins and MD was dominated by hydrogen bonds and hydrophobic interactions. These components also contributed to the construction of a Glu-scaffolded network, enabling the SPI-WGP-MD gel system to form a three-dimensional interpenetrating grid structure. This configuration increased cross-linking sites and strengthened the gel network’s structural integrity. However, the excessive addition of MD competed with SPI and Gli for water. This led to grid fracture and “phase separation”, and then the pores became larger, thereby reducing the ability of the gel network to retain water and weakened the gelation behavior of the system.

## 3. Conclusions

This study validated the feasibility of MD in improving the gel properties of SPI-Glu/Gli-MD composite gels by varying the Glu/Gli ratio and MD dosage. Experimental results show that the high-Gli group exhibits superior gel strength, water retention, hydrogen bonding, and β-sheet content, while the high Glu group demonstrates higher S-S and hydrophobic interactions, stronger fluorescence intensity, and confers greater elasticity to the composite gel. Intermolecular force analysis revealed that MD addition shifted the dominant forces in the gel system from hydrophobic interactions and S-S bonds to hydrogen bonds and hydrophobic interactions. Furthermore, FTIR, endogenous fluorescence spectrum, and SEM analyses indicated that MD promoted a further increase in the β-sheet content of the SPI-WGP composite gel while reducing fluorescence intensity. MD was cross-linked with Gli and SPI and filled the mixed network structure—primarily Glu-based—with aggregates, forming a supported, dense, and uniform gel system. Compared to the single SPI control group gel, the composite gel exhibited superior WHC, gel texture, and microstructure at a Glu/Gli ratio of 4:6 and 4% MD content. Excessive MD, however, disrupted the interaction between proteins and led to phase separation of the composite gel. These findings provide theoretical support for optimizing the SPI-WGP-MD gel system as a structural matrix for the application of plant-based meat.

## 4. Materials and Methods

### 4.1. Material and Reagents

WGP (85.1% protein content) was purchased from Shandong Qufeng Food Technology Co., Ltd. (Anqiu, China). SPI (91.9% protein content) was purchased from Harbin Xichi Biotechnology Co., Ltd. (Harbin, China). Maltodextrin (food grade) was sourced from Shandong Jinan Wangxi Group Co., Ltd. (Shandong, China). DTNB reagent was purchased from Shanghai Aladdin Biochemical Technology Co., Ltd. (Shanghai, China, Lot#J2226384). All reagents used in this study were of analytical grade.

### 4.2. Extraction of Glu and Gli

The extraction of Gli and Glu was based on the ethanol extraction method by Li et al. [51]. According to a solid–liquid ratio of 1:20 (g/mL), 65% ethanol was added to the WGP, followed by magnetic stirring (EMS-9A, Aurora, Tianjin, China) at 25 °C for 3 h. The mixture was then centrifuged (L2-4K, Kecheng, Changsha, China) at 1699× *g* for 10 min, and the supernatant was collected. The 65% ethanol was added to the precipitate for repeated extraction, and the supernatants were combined and diluted. The pH of the combined supernatant was adjusted to 6.2, after which the mixture was allowed to stand and dried to obtain Gli. The precipitate was collected and washed with water to remove residual ethanol, and Glu was obtained after drying. The dried Glu and Gli were crushed and sieved through an 80 µm mesh sieve.

### 4.3. Preparation of Mixed Gel Samples with Different Ratios of Glu/Gli and MD Additions

The method was slightly modified with reference to Qu et al. [12], Li et al. [51], and Xie et al. [15]. The extracted Glu and Gli were mixed at different ratios (0:10, 3:7, 4:6, 5:5, 6:4, 7:3, and 10:0). WGP dispersions were prepared with distilled water and stirred at 25 °C and 600 rpm for 10 min using a magnetic stirrer (EMS-9A, Tianjin Ono Instruments Co., Ltd., Tianjin, China). After sufficient water absorption, SPI was added at an SPI–Glu/Gli ratio of 7:3, and the mixture was stirred uniformly to obtain an SPI-Glu/Gli composite solution with a total protein content of 16% (*w*/*w*). The protein addition was kept constant. MD was added to the SPI-WGP composite mixture at 0, 2, 4, and 6%, and mixed evenly. Then, it was placed in a water bath pot (DK-8D, Yiheng, Shanghai, China) and heated at 95 °C for 30 min. The SPI-WGP composite mixture was cooled in an ice water bath and placed in a refrigerator at 4 °C overnight to obtain the SPI-WGP composite gel. After freeze drying, the SPI-WGP composite gel was crushed through an 80 µm mesh sieve and set aside. The 16% (*w*/*w*) SPI gel was set as the control group.

### 4.4. Gel Strength and Textural Properties

The gel strength of SPI-WGP composite gel was measured using a Texture Profile Analyzer (TA-XT2i, SMS, London, UK). The sample was placed on the measuring table for determination using a P/0.5 probe. Experimental parameters were set as pre-test speed of 2.0 mm/s, mid-test speed of 1.0 mm/s, and post-test speed of 1.0 mm/s. The trigger force was 5.0 g, the number of test cycles was 1, and the test distance was 10 mm. The maximum trigger force was used as the gel strength value.

The hardness, elasticity, chewiness and cohesiveness of the SPI-WGP composite gels were measured using a Texture Profile Analysis (P50 probe). The gel samples were placed in the center of the test bench. The parameters were as follows: pre-test speed of 2.0 mm/s, test rate of 1.0 mm/s, and post-test rate of 1.0 mm/s. Trigger force was 5.0 g and cycled twice.

### 4.5. Rheological Properties

The rheological properties of the SPI-WGP composite gels were achieved using the method of Niu et al. [52] with minor modifications. The effect of the thermal gelation process on the rheological properties of the mixed SPI-WGP composite gel were measured using a rheometer (MCR102, Anton Paar, Graz, Austria) within a strain range of 0.01% to 100% at 25 °C and 1 Hz. The dynamic measurement mode was adopted, probe diameter was 40 mm, gap distance was 1.8 mm, and the mode was oscillating. Frequency scanning measurement: The temperature was constant at 25 °C, strain was set to 1%, and the frequency varied in the range of 0.1–10 Hz.

### 4.6. WHC

WHC of the SPI-WGP composite gels was determined using the method of Du et al. [53] with slight modification. Gel sample (2 g, W_1_) was centrifuged at 4 °C and 8000 r/min (TG16-WS, BioRidge, Shanghai, China) for 15 min. The centrifuged sample was transferred to the filter paper to remove moisture and was weighed (W_2_). The calculation formula for *WHC* is as follows:

(1) $$WHC(\%)=\frac{W_{2}}{W_{1}}\times 100$$

In the formula, W_1_ is the mass of the sample before centrifugation, and W_2_ is the mass of the sample after centrifugation and removal of the spilled water.

### 4.7. Intermolecular Forces

#### 4.7.1. Detection of S-S Content

The S-S content in the SPI-WGP composite gels was determined using the method of Hu et al. [50] with slight modification. Two portions of 15 mg freeze-dried samples were weighed, respectively. One portion was added to 5 mL Tris-Gly buffer, and the other was added to 5 mL Tris-Gly-8m Urea solution. After mixing evenly, 50 μL DTNB (4 mg/mL) was added, respectively. The mixture was kept in the dark at 25 °C for 1 h and centrifuged at 8000 r/min for 10 min. The absorbance of the supernatant was measured at 412 nm using an ultraviolet spectrophotometer (UV 5100B, Yuanxi, Shanghai, China). Solution without the added sample was used as a blank. Each sample was subjected to three parallel experiments. The free and total sulfhydryl (SH) content and S-S content are calculated as follows [54]:

(2) $$SH(\mu \mathrm{mol}/g)=\frac{73.53\times A_{412\mathrm{nm}}}{C}$$

(3) $$SS(\mu \mathrm{mol}/g)=\frac{\mathrm{Total}\mathrm{SH}\mathrm{content}-\mathrm{Free}\mathrm{SH}\mathrm{content}}{2}$$

In the equation, 73.53 = 10^6^/(1.36 × 10^4^), 1.36 × 10^4^ is the molar absorption coefficient of DNTB; A_412nm_ is the absorbance at 421 nm; and C is the protein concentration of the sample, mg/mL.

#### 4.7.2. Detection of Non-Covalent Bond Content

The non-covalent bond content in the SPI-WGP composite gels was determined using Cao et al. [55] for a slightly modified approach. The four phosphate buffers used to dissolve the freeze-dried samples are as follows: 0.05 moL/L NaCl (PA); 0.6 moL/L NaCl (PB); 0.6 moL/L NaCl + 1.5 moL/L urea (PC); and 0.6 moL/L NaCl + 8 moL/L urea (PD). Sample powder (3 g) was dissolved in 20 mL of the above four phosphate buffer solutions and homogenized for 5 min, then centrifuged at 8000 r/min for 15 min. The supernatant was decanted, and the protein concentration of the supernatant was determined by the Komas Brilliant Blue method. The ionic bond contribution was expressed as the difference in protein content dissolved in PB and PA. Hydrogen bond contribution was expressed as the difference in protein content dissolved in PC and PB, and the hydrophobic interaction contribution was expressed as the difference in protein content dissolved in PD and PC.

### 4.8. Intrinsic Fluorescence Spectroscopy

According to the method of Zhao C et al. [18], the fluorescence spectrum of the SPI-WGP composite gels was determined with a fluorescence spectrometer (F-7000, Hitachi, Tokyo, Japan). The SPI-WGP composite gel-lyophilized powder sample was dispersed in phosphate buffer (0.01 mol/L, pH 7.0) to obtain a solution of 1.5 mg/mL. The excitation wavelength was set at 290 nm, the emission spectrum scanning range was 300–450 nm, and the slit width was 5 nm.

### 4.9. FTIR

The freeze-dried protein samples were evenly covered at the sample port of the Fourier infrared detector (PerkinElmer, Waltham, MA, USA). The scanning range was 4000–400 cm^−1^, resolution was 4 cm^−1^, and the number of scans was 32. The relative contents of the secondary structures were analyzed using Peakfit 4.12 software. For the amide I band (1600–1700 cm^−1^) in the infrared spectrum, baseline correction, smoothing, deconvolution, and second-derivative fitting were performed in turn, and the relative contents were calculated by quantifying the integrated area of each corresponding sub-peak [56].

### 4.10. SDS-PAGE

SPI-WGP composite gels were subjected to SDS-PAGE using the method of Jia et al. [56] with modifications. The freeze-dried protein sample was diluted with 0.8% urea to 80 mg/mL, 20 μL of the supernatant was drawn into a centrifuge tube, and 5 μL of reduced loading buffer was added. The mixture was homogenized, boiled in water bath for 5 min, and, after cooling, was centrifuged at 10,000 r/min for 10 min. The loading volume was 10 μL (marker loading volume was 10 μL), and electrophoresis was performed at a voltage of 160 V. The sample was run to 1 cm from the bottom to stop electrophoresis, and the gel was taken out, stained, and decolorized. The electrophoretogram was analyzed by Quantity-One 4.6.2 software.

### 4.11. SEM

The surface structure of the SPI-WGP composite gels were studied using SEM based on the method of Chen et al. [57] with modifications. The gel was cut into slices, fixed with glutaraldehyde at pH 6.8 for 6 h, and then frozen at −80 °C for 12 h. After freeze drying, the samples were sprayed with gold, and the images were observed using a scanning electron microscope (S-3400N, Hitachi, Japan) with an acceleration voltage of 20 kV and a magnification of 2000×.

### 4.12. Statistical Analysis

All measurements were carried out in triplicate (*n* = 3). All data were analyzed using SPSS 23.0 to determine significant differences based on one-way ANOVA and Duncan’s multiple comparison test at *p* < 0.05. Results were presented as mean ± standard deviation (SD). OriginPro 2021 was used for plotting the graphs.

## Data Availability

The original contributions presented in the study are included in the article; further inquiries can be directed to the corresponding author.

## Supplementary Materials

The following supporting information can be downloaded at: https://www.mdpi.com/article/10.3390/gels11110916/s1. File S1: Quantitative data of intermolecular forces (Section 2.4).
