# Supplementary material for: Impact of Glutenin/Gliadin Ratio and Maltodextrin on Structural and Functional Properties of Soy Protein Isolate–Wheat Gluten Protein Composite Gel
Source: Gels. 2025 Nov 16;11(11):916. doi: 10.3390/gels11110916 (PMC12651960; doi:10.3390/gels11110916)
Supplement: Supplementary file 1 [file gels-11-00916-s001.zip › gels-3971427-supplementary.pdf]

Supplementary Material

Quantitative Results of Intermolecular Forces

When the maltodextrin (MD) addition level was 4%, the group with a glutenin/gliadin (Glu/Gli) ratio of 4:6 exhibited the highest hydrogen bond content (43.69 μg/g), representing a 153.5% increase compared to the single soy protein isolate (SPI) control group gel. At an MD addition level of 4%, the group with a Glu/Gli ratio of 6:4 achieved the maximum hydrophobic interaction (48.16 μg/g), which was an 83.3% enhancement relative to the single SPI control group gel. When MD was excessive (6%), the ionic bond content in the group with a Glu/Gli ratio of 10:0 reached the minimum value (1.43 μg/g), a decrease of 86.7% compared to the single SPI control group gel. The free sulfhydryl (-SH) content was the lowest (15.35 μg/g) in the group with a MD addition level of 6% and a glutenin/gliadin (Glu/Gli) ratio of 6:4, which was a 42.4% decrease compared to the single SPI control group gel.
